# Applications of different machine learning approaches in prediction of breast cancer diagnosis delay

**DOI:** 10.3389/fonc.2023.1103369

**Published:** 2023-02-16

**Authors:** Samira Dehdar, Khodakaram Salimifard, Reza Mohammadi, Maryam Marzban, Sara Saadatmand, Mohammad Fararouei, Mostafa Dianati-Nasab

**Affiliations:** ^1^ Computational Intelligence & Intelligent Optimization Research Group, Business and Economic School, Persian Gulf University, Bushehr, Iran; ^2^ Business Analytics Section, Amsterdam Business School, University of Amsterdam, Amsterdam, Netherlands; ^3^ Department of Public Health, School of Public Health, Bushehr University of Medical Science, Bushehr, Iran; ^4^ Department of Epidemiology, School of Public Health, Shiraz University of Medical Sciences, Shiraz, Iran; ^5^ Department of Complex Genetics and Epidemiology, School of Nutrition and Translational Research in Metabolism, Maastricht University, Maastricht, Netherlands

**Keywords:** breast cancer (BC), random forest (RF), neural networks (NN), delay, machine learning, extreme gradient boosting, logistic regression

## Abstract

**Background:**

The increasing rate of breast cancer (BC) incidence and mortality in Iran has turned this disease into a challenge. A delay in diagnosis leads to more advanced stages of BC and a lower chance of survival, which makes this cancer even more fatal.

**Objectives:**

The present study was aimed at identifying the predicting factors for delayed BC diagnosis in women in Iran.

**Methods:**

In this study, four machine learning methods, including extreme gradient boosting (XGBoost), random forest (RF), neural networks (NNs), and logistic regression (LR), were applied to analyze the data of 630 women with confirmed BC. Also, different statistical methods, including chi-square, p-value, sensitivity, specificity, accuracy, and area under the receiver operating characteristic curve (AUC), were utilized in different steps of the survey.

**Results:**

Thirty percent of patients had a delayed BC diagnosis. Of all the patients with delayed diagnoses, 88.5% were married, 72.1% had an urban residency, and 84.8% had health insurance. The top three important factors in the RF model were urban residency (12.04), breast disease history (11.58), and other comorbidities (10.72). In the XGBoost, urban residency (17.54), having other comorbidities (17.14), and age at first childbirth (>30) (13.13) were the top factors; in the LR model, having other comorbidities (49.41), older age at first childbirth (82.57), and being nulliparous (44.19) were the top factors. Finally, in the NN, it was found that being married (50.05), having a marriage age above 30 (18.03), and having other breast disease history (15.83) were the main predicting factors for a delayed BC diagnosis.

**Conclusion:**

Machine learning techniques suggest that women with an urban residency who got married or had their first child at an age older than 30 and those without children are at a higher risk of diagnosis delay. It is necessary to educate them about BC risk factors, symptoms, and self-breast examination to shorten the delay in diagnosis.

## 1 Introduction

Breast cancer (BC), the most frequently diagnosed cancer (1) and the second leading cause of death among women (2), accounts for nearly 35% of new cancer cases (3). In 2021, BC was recognized as the leading cause of mortality among women all over the world, with more than 685,000 deaths and 2.3 million new cases, equivalent to 11.7% of all identified cancer cases (1), causing 15% of all cancer deaths, mainly in less-developed countries (4).

Specifically, developing countries are suffering from an increasing number of BC cases with an increasing range of young women at risk of cancer (5). In recent years in Asian countries, including Iran, both the incidence and mortality of BC have had notable growth (6–9). Also, studies have declared that the average age of BC in Iranian women is almost a decade earlier than the world average (10, 11). Also, in Iran, delays in diagnosis and treatment (12, 13) and cancer detection at more advanced stages compared to Western countries have been reported (14).

The prolonged interval from the detection of initial symptoms until the histological diagnosis is defined as a diagnosis delay (15), which might happen for two main reasons: 1) patients’ delay, which refers to the duration between noticing the first symptom and announcing it to the medical consultant, and 2) providers’ delay, which is identified as the time interval between the first announcement of symptoms to the start of treatment (16). Longer delays lead to more advanced stages of cancer (17) and consequently a lower chance of survival (18, 19). Clinically, a 90-day or more delay in diagnosis is considered a delayed BC diagnosis (20).

Several studies have found that various factors are associated with BC diagnosis delays. Effective sociodemographic factors include age (21), education (22, 23), socioeconomic status (24, 25), marital status (22, 26), place of residence (27, 28), and family history (26, 29). Other important factors for a delayed presentation that lead to diagnosis delay are lack of knowledge regarding the disease (25, 30), lack of breast self-examination (23, 28), ignorance (25, 26, 31), stress of cancer treatment and consequences (32), and absence of qualified healthcare service (30, 31).

Machine learning, a subfield of artificial intelligence, uses a wide range of optimization, probabilistic, and statistical methods that allow computers to “learn” from past examples and to distinguish hard-to-detect patterns from complicated datasets. In the medical field, clinics and hospitals record and keep massive databases of patients’ symptoms and diagnoses. Therefore, researchers use this knowledge to develop classification models that can make inferences based on historical cases (33).

This study aimed to analyze the importance of a variety of factors to predict BC diagnosis delay by employing four different machine learning methods, including random forest (RF), neural network (NN), logistic regression (LR), and extreme gradient boosting (XGBoost).

## 2 Materials and methods

**Figure 1:**
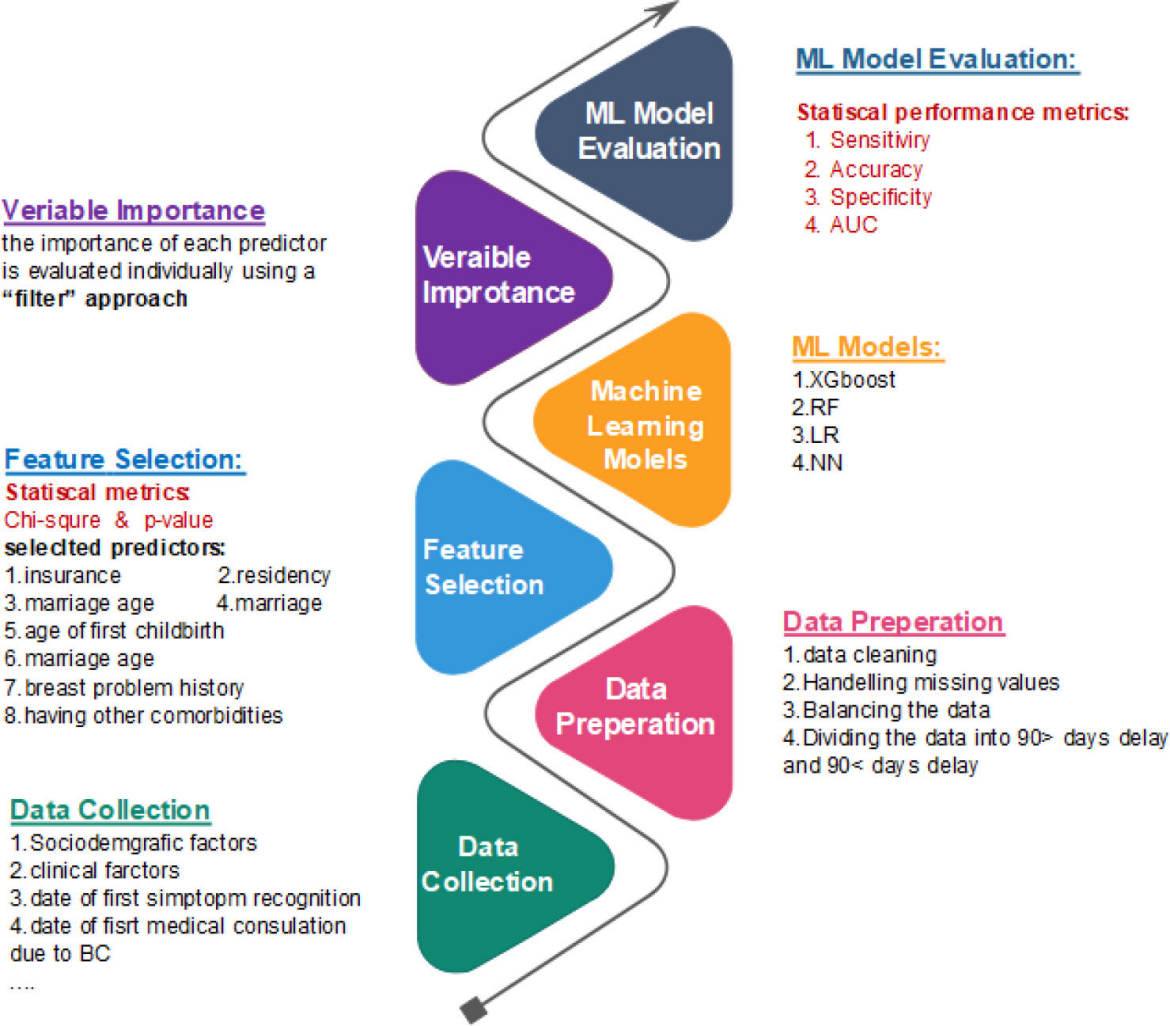
Steps for building the prediction model and the statistical methods used in each step.

In this study, a six-step methodology was applied to build a prediction model. **Figure 1** illustrates an overview of the steps taken and the statistical methods that were used in each step. Different statistical methods, including chi-square, p-value, sensitivity, specificity, accuracy, and area under the receiver operating characteristic (ROC) curve (AUC), were utilized in this paper.

### 2.1 Data

In this study, 630 women with confirmed BC (incident or new cases) were assessed to identify the factors related to delayed diagnosis of BC. The data were obtained partly from the patients’ hospital records and partly from an interview-administered questionnaire that was completed during the study period while the patients were visiting the center. Literate patients read and gave signed informed consent. Verbal consent was obtained from illiterate patients. Ethical approval was obtained from the Shiraz University of Medical Sciences ethics committee (23). A trained nurse was hired to interview the patients by using a validated questionnaire (23). The questionnaire and interview procedures were evaluated and revised during a pilot study on 50 patients. Accordingly, using the test–retest method, the questionnaire’s reliability was estimated to be good (Cronbach alpha = 0.76) (23). Furthermore, other data, including self-reported date or type of initial signs and symptoms of BC noticed by the patients, date of first symptom recognition, and the month and year of their first medical consultation due to BC, were also collected. These dates were used as a reference to questions about whether or not the patients had perceived symptoms, the period before the first consultation, and socioeconomic factors at the moment of the first medical consultation. Even though a standard questionnaire was used to collect both clinical and sociodemographic factors, some factors were put aside due to the missing data (such as body mass index (BMI) and menopause status).

Patients were divided into two categories: those 1) with less than 90 days’ delay in diagnosis and 2) with more than 90 days’ delay in diagnosis. Different features were analyzed in both groups, including age, marriage, residency, insurance, age at first childbirth, marriage age, having other comorbidities, and other breast disease histories. The main reason for the delay in diagnosis was also obtained from patients. In the second phase, clinical data including the stage of disease, tumor size, and lymph node status, was gathered by reviewing patients’ medical records (23). In this study, patients’ age was considered a continuous variable. The age at first marriage was divided into five categories (20, 20–25, 25–30, >30, and not married), and the age at first childbirth was divided into four (20, 20–25, > 30, single, or not having a child). Both sociodemographic and clinical data are shown in **Table 1**.

**Table 1:** Sociodemographic and clinical factors.

Sociodemographic data	
Age	Year
Education	Primary and lower, middle school, high school, college
Age at first marriage	Year
Marital status	Single, married
Occupation	Employed, housewife
Menopausal status	Pre-menopausal, post-menopausal
Residency	Rural, urban
Health insurance	Yes, no
Daily exercise	<10, 10–20, >20 min
BMI (kg/m^2^)	Underweight, normal, overweight, obese
Smoking	Yes, no
X-ray history	Yes, no
Chronic disease	Yes, no
Delay time	Day
Family history of BC	Yes, no
Age at first pregnancy	Year
History of BD	Yes, no
Status of knowledge and regular practice of BSE	Yes, no
Clinical data	
Type of first symptom	Lump, discharge, pain, and others
Location of tumor	Right, left
Tumor type	Ductal, lobular/medullary, and others

BMI, body mass index; BD, breast disease; BSE, breast self-examination.

### 2.2 Machine learning methods

To optimize the hyperparameters for all the algorithms (RF, NN, XGBoost, and LR) in the train set, the grid search method in the Caret package (Kuhn, 2008) in the R programming language was used. **Table 2** shows the parameter values for each applied machine learning (ML) algorithm.

**Table 2:** Parameter values of the four applied ML algorithms.

Algorithm	Parameter	Value/setting
LR	Fitting method	Iteratively reweighted least squares
NN	Hidden layer	1
	Input layer	1
	Output layer	1
	Fitting method	Entropy
	Maximum number of iterations	100
	Maximum number of weights	1,000
RF	Number of trees to grow	200
	Minimum size of terminal nodes	1
XGBoost	Max depth	1
	Number of rounds	150
	Minimum child weight	1
	Eta	0.3
	Subsample ratio of columns	0.8
	Subsample	0.5

ML, machine learning; LR, logistic regression; NN, neural network; RF, random forest; XGBoost, extreme gradient boosting.

#### 2.2.1 Random forest

The RF algorithm is known as a highly stated machine learning method for classification problems (33). The algorithm has been reported to originate one of the greatest accuracies (34). Computing the missing data and investigating multi-dimensional data are possible by RF algorithm (35). The significance of variables used for classification in RF can also be tuned in (35). The RF is a combined classification method based on the decision tree model. K decision trees are generated based on K diverse training data extracted from the main dataset. Decision trees build the final RF model (36). In such combined methods as RF, a “‘strong learner” is constructed by consuming numerous “weak learners” (37).

In this paper, to make the parameters appropriate for using the RF method, the number of trees was set to 200, and the minimum size of terminal nodes was set to one.

#### 2.2.2 Logistic regression

**Figure 2:**
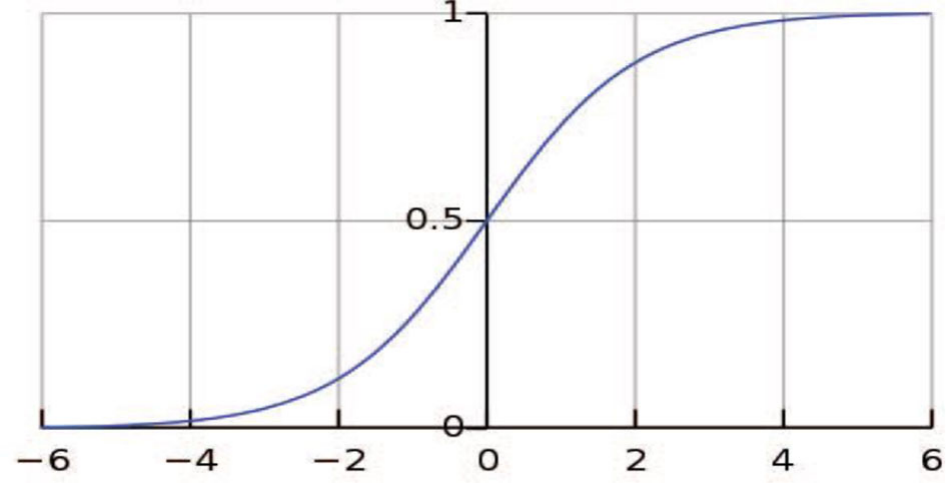
Logistic regression curve.

Utilizing binary variables for classification problems can be performed by LR. This model generally demonstrates the probability of an event occurrence by measuring the correlation between a dependent binary variable and a minimum of one independent variable (38). The distribution of the odds is outlined in an S-shaped function (**Figure 2**) to achieve an output between 0 and 1 (39). As LR is mathematically bound to generate probabilities in the range of [0, 1], in case values are below 0.5, they will be assumed as 0; otherwise, they will be considered 1 (40).

The logistic function is shown in Equation 1:


(1) $$S\left(z\right)=\;\frac{1}{1+\;e^{-z}},$$

where *S*(*z*) represents the probabilities in the range of [0, 1], *z* is the input, and *e* is a natural constant (41). In this paper, a multivariable LR with 20 predictors was used to define factors affecting BC diagnosis delay. The iteratively reweighted least-squares method was applied to make this method fit the available data (42).

#### 2.2.3 Neural networks

The NN method is used in a vast variety of issues as a result of its superior implementation in classification problems. NN is one of the most reputable machine learning algorithms (43). This method is inspired by biological neural networks (44). The NN method is made up of a three-layered feedforward network. The notion of weights among hidden layers, the output–input layer in the network, leads to learning (45). The output of a neuron in NN achieves in two steps, using the following formulas (46):

Step 1: *x_ij_* stands for the *i*th input to node *j*, and *W_ij_* indicates the weight related to the *i*th input to node *j*.


(2) $$\sum_{i}W_{ij}x_{ij}.$$

Step 2: *e* is a natural constant, and *x* is the input of the function.


(3) $$y=\;\frac{1}{1+\;e^{-x}}.$$

In this study, this method was utilized by setting one input layer including 20 variables, a hidden layer, and one output layer. The entropy fitting method was used to fit the NN to the dataset. The maximum number of iterations and the maximum number of weights were set to 100 and 1,000, respectively.

#### 2.2.4 Extreme gradient boosting

XGBoost is a powerful boosting algorithm in the machine learning system (33). XGBoost is a kind of regression tree capable of supporting both regression and classification. XGBoost and decision trees have similar decision-making rules (47). With the use of an appropriate data structure, the XGBoost algorithm is able to optimize, predict, and classify a system with the highest accuracy (19). This algorithm organizes the data to reduce the lookup time to a minimum. It also leads to cutting down the model’s training time and, at the same time, improves the accuracy of the classification (48). The XGBoost algorithm is thriving as a result of its high scalability in any type of scenario (49).

In this paper, the number of rounds was set to 150 with a max depth of 1, an eta of 0.3, and a minimum child weight of 1. The subsample ratio of columns was considered to be 0.8, and the subsample was 0.5.

### 2.3 Feature selection

Feature selection is a practical, data-filtering evaluation procedure (50). In feature selection strategies, a subset of features from the primary dataset is picked by evaluating the relevance of the data to show inter-group impacts (51). Feature selection is not dependent on any machine learning algorithms. Instead, features are selected on the basis of their scores in various statistical tests for their correlation with the outcome variable. Chi-square is a statistical test applied to groups of categorical features to evaluate the likelihood of correlation or association between them using their frequency distribution.

To decide which features must be taken into consideration in building the prediction model, chi-square was calculated for 20 variables. Seven variables, including insurance, residency, marriage age, age of first childbirth, marriage, breast problem history, having other comorbidities, and marriage age, were chosen as the machine learning targets. For age, as the only continuous variable in the dataset, a p-value was calculated, so age is considered the eighth selected feature to construct the prediction model. The outcome of evaluating the chi-square for variables is shown in **Table 3**.

**Table 3:** Outcome of chi-square method of selected variables.

Variables	Insurance	Residency	Marriage age	Age of first childbirth	Marriage age	Marriage	Breast problem history	Other comorbidities
**p-Value**	0.046	0.023	0.038	P< 0.005	0.038	0.006	0.009	**0.007**

Insurance (p-value: 0.046), residency (p-value: 0.023), marriage age (p-value: 0.038), marital status (p-value: 0.006), breast problem history (p-value: 0.009), and having other comorbidities (p-value: 0.009) were found to be BC delay predictive factors when utilizing the chi-square method, and other features including patient or doctor delay (0.42), tumor type (0.41), location of the tumor (left/right breast) (0.11), first symptoms (0.93), education (0.07), income (0.10), job (0.52), family history (0.38), awareness of breast self-examination (0.20), daily exercise (0.19), chest X-ray history (0.07), and smoking (0.07) where omitted after measuring the amount of chi-square (higher than 0.05).

### 2.4 Variable importance

The importance of each predictor is evaluated individually using a “filter” approach. The filter method ranks each feature based on some univariate metrics and then selects the highest-ranking features. In this study, age was found to be of the highest importance in all methods conducted. Putting age aside, urban residency was the most effective variable in the RF and XGBoost methods, while in the NN method, it was found to be the least important one. Despite the fact that insurance is expected to increase patients’ willingness to attend doctor appointments and undergo mammography, preventing delayed diagnosis, it has gained a low level of importance in all methods. Variables of importance in the four ML models are shown in **Table 4**.

**Table 4:** Variable importance.

Variable importance				
	XGBoost	RF	NN	LR
Age	100	100	100	100
Urban residency	17.54	12.04	0.63	43.19
Ever married	5.276	0.42	50.05	<0.001
Marriage age (20–25)	6.81	6.01	5.28	33.20
Marriage age (25–30)	3.65	3.32	15.09	38.90
Marriage age (>30)	2.32	0.71	18.03	34.73
Nulliparous	4.71	5.11	14.24	44.19
Age at first childbirth (20–30)	13.13	8.88	0.14	31.30
Age at first childbirth (>30)	11.42	6.80	15.36	82.57
Other breast disease history	12.37	11.58	15.83	42.99
Having other comorbidities	17.14	10.72	6.34	49.41
Health insurance	3.01	4.37	<0.001	14.61

XGBoost, extreme gradient boosting; RF, random forest; NN, neural network; LR, logistic regression.

## 3 Results

Among 630 BC patients, 204 (32%) had a diagnosis delay of more than 90 days. Among patients with a diagnosis delay of more than 90 days, 29.90% were between 40 and 50 years old, 88.72% were ever married, and 72.05% had urban residency. Only 15.19% of patients in this category did not have insurance, 52.45% were married when they were younger than 20 years, and 35.78% had given birth to their first child before they were 20 years old.

Among 426 patients who had a diagnosis delay of fewer than 90 days, 35.21% were between 40 and 50 years old, 54.47% were married at an age younger than 20 years, and 43.90% had their first experience of childbirth when they were younger than 20 years; 84.27% had a history of other breast comorbidities, and 80.75% had urban residency. The study population is shown in **Table 5**.

**Table 5:** Statistics of BC patients based on model variables.

Variables		Delay in diagnosis (<90 days) (n = 426) (68%)	Delay in diagnosis (>90 days) (n = 204) (32%)	Total (n = 630)
**Age (years)**	<40	110 (25.82%)	51 (25.00%)	161 (25.56%)
	40–50	150 (35.21%)	61 (29.90%)	211 (33.49%)
	50–60	113 (26.52%)	56 (27.45%)	169 (26.83%)
	>60	53 (12.44%)	36 (17.64%)	89 (14.12%)
**Marriage**	Single (never married)	22 (5.16%)	23 (11.27%)	45 (7.14%)
	Ever married	405 (94.84%)	181 (88.72%)	585 (92.86%)
**Place of residence**	Rural	81 (19.25%)	57 (27.94%)	139 (22.06%)
	Urban	344 (80.75%)	147 (72.05%)	491 (77.94%)
**Insurance**	No	39 (9.15%)	31 (15.19%)	70 (11.11%)
	Yes	387 (90.85%)	173 (84.80%)	560 (88.89%)
**Age at first childbirth**	<20	187 (43.90%)	73 (35.78%)	260 (41.27%)
	20–30	156 (36.62%)	56 (27.45%)	212 (33.65%)
	>30	36 (8.45%)	37 (18.13%)	73 (11.59%)
	Single or no child	47 (11.03%)	38 (18.62%)	85 (13.49%)
**Marriage age**	<20	232 (54.47%)	107 (52.45%)	339 (53.81%)
	20–25	94 (22.06%)	37 (18.13%)	131 (20.79%)
	25–30	56 (13.14%)	24 (11.76%)	80 (12.70%)
	>30	23 (5.40%)	14 (6.86%)	37 (5.87%)
	Not married	21 (4.93%)	22 (10.78%)	43 (6.83%)
**Other comorbidities**	No	280 (65.73%)	111 (54.41%)	391 (62.06%)
	Yes	146 (34.27%)	93 (45.59%)	239 (37.94%)
**Other breast disease history**	No	359 (84.27%)	154 (75.49%)	513 (81.42%)
	Yes	67 (15.73%)	50 (24.51%)	117 (18.57%)

BC, breast cancer.

### 3.1 Evaluation metrics

Different performance measures were utilized to analyze each indicator’s importance in delayed BC diagnosis, as described in this part. Specificity, sensitivity, and ROC curves are commonly used in binomial classification tests to measure the performance of the statistics. The proportions of negatives are scaled by “specificity”, while the extent of actual positives is scaled by “sensitivity”. The specificity and sensitivity are calculated by Equations 4 and 5, respectively.


(4) $$Specificity=\frac{TN}{\left(TN+FP\right)},$$

(5) $$Sensitivity=\frac{TP}{\left(TP+FN\right)},$$

where *TP* means true-positive rate; *TN*, true-negative rate; *FP*, false-positive rate; and *FN*, false-negative rate.

The performance measures for the four machine learning methods are reported in **Table 6**. As shown, LR has the best performance in terms of accuracy, while NN, LR, and XGBoost have been able to have more considerable sensitivity.

**Table 6:** Performance measures of four ML models.

Measure	RF	NN	LR	XGBoost
Accuracy	0.6967	0.7213	0.729	0.7131
Sensitivity	0.8372	0.8721	0.8721	0.8721
Specificity	0.3611	0.3611	0.3889	0.3333
AUC	0.788	0.765	0.715	0.688

ML, machine learning.

**Figure 3:**
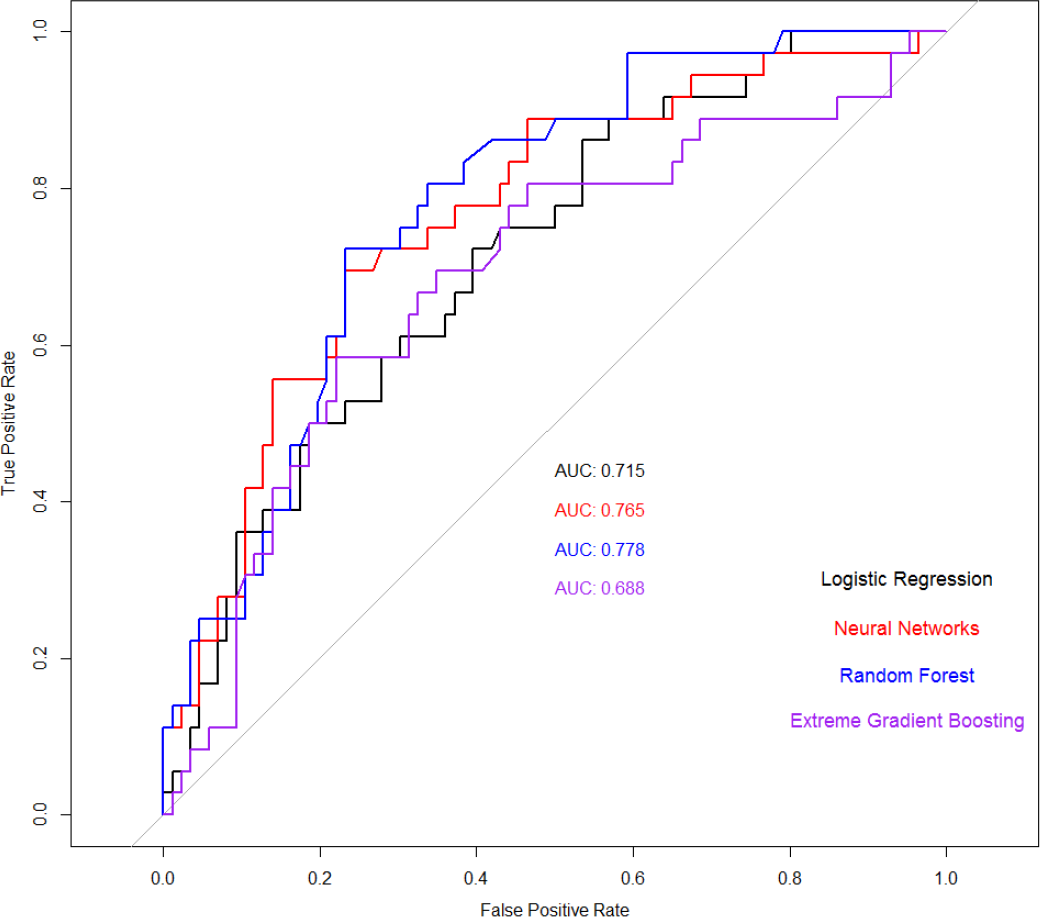
Receiver operating characteristic (ROC) curves of four applied machine learning (ML) models and the area under the curve (AUC) are specified for each model.

AUC shows how qualified a parameter is at discerning among a couple of diagnostic categories. **Figure 3** illustrates a comparative analysis of four different classification methods on the ROC curve. According to the ROC curve, RF has the highest AUC, while NN and LR have the second and third highest AUC, respectively.

## 4 Discussion

The results show 32% of patient delay among women in Iran, which is a moderate amount in comparison with that in other developing countries, such as Pakistan (88.8%) (52), Uganda (89%) (53), Nigeria (81.6%) (26), and China (34%) (53). However, in developed countries, the situation is quite different. In the USA, the patient delay was reported to be 17.5% in white patients and 26.4% in African American patients (52). In the UK, 8.4% of BC patients postponed looking for treatment for more than 3 months (54), and in Malaysia, the patient delay was reported to be 33.1% (50). Therefore, compared to the reported amount in surveys from developed countries, the current study showed a more intense patient delay.

In this study, four machine learning methods, including XGBoost, RF, NN, and LR, were applied to analyze the variables’ importance. In all methods, “age” was found to be of the highest importance. Putting age aside, urban residency (17.54), having other comorbidities (17.14), and age at first childbirth (>30) (13.13) were found to be the top three important variables in the XGBoost method. In the RF method, the outcome was almost identical to the XGBoost method, where the top three essential predictors (leaving “age” out) were urban residency (12.04), other breast disease history (11.58), and having other comorbidities (10.72). Conducting the NN method, being married (50.05), marriage age (>30) (18.03), and other breast disease history (15.83) were found to be the top three effective risk factors. Considering the top three important predictors in the LR method, the only factor in common with the RF and XGBoost methods was having other comorbidities (49.41). With the use of this method, the outcome highlighted the first childbirth age, the age at the first childbirth at >30 (82.57), and being nulliparous (44.19) as the top three among the study variables.

In a study by Mirfarhadi et al. (55), 232 patients with confirmed BC in Iran were studied, and LR was applied to identify the main risk factors for BC diagnosis delay. Among the 16 factors that were studied in this paper, including age, place of residence, education level, marital status, number of children, monthly income, having insurance coverage, having complementary insurance, family history of BC, history of mammography, and stage of disease, the most important factors were found to be the stage of disease, primary insurance, and lack of complimentary insurance. Passing over the stage of disease and history of mammography, other factors were similar to the current study, whereas the same method “LR” showed a completely different outcome. Implementing the LR method in the current study, age, age at first childbirth, and having other comorbidities were found to be the most important factors in BC delayed diagnosis. In the analysis of 283 women with BC, taking similar factors such as age, place of residence, education level, medical payment method (insurance), monthly income, method of symptom discovery, knowledge of BC symptoms, family support, health values, internal and external health locus of control, and perceived health competence into consideration, the main BC delay predictors announced were knowledge of BC symptoms, external health locus of control, breast self-examination/physical examination, perceived health competence, family support, pain stimulation, and age.

In Senegal, data collected from patients within 7 years was studied (56) to analyze the association between sociodemographic factors and BC delay. In this study, no associations were detected between sociodemographic factors and BC delay, and the only relevant factor was found to be a negative history of family BC. In the UK (57) and Malaysia (58), which are also known as developed countries, the most important sociodemographic factor correlated to BC delay risk was found to be “marital status”, as reported in (56, 59), and married women had a shorter delay than single and separated/divorced women. The results show that in developed countries, socioeconomic factors have little effect on the risk of delayed BC diagnosis. This can be a result of governmental planning and support, something that is not actually seen in less-developed countries. In a study in China (60), 1,431 women with diagnosed BC were studied to assess the correlation between variables including demographic data, clinical and tumor characteristics, and BC delay by employing multivariate LR and Kaplan–Meier regression models, and it was directly reported that there was no association between age and BC delay. In contrast, 7 years later, another study (61) in the same country declared age as the main factor affecting BC diagnosis delay. In this study, multiple linear regression was utilized to measure the impact of sociodemographic characteristics, medical history, and knowledge of BC; residency and disclosure of symptom were the most important factors, excluding age as the vital factor. In another developing country, Ethiopia, age was declared as the main factor correlating with BC diagnosis delay (25). In this study, bivariable and multivariable LRs were conducted to assess the prevalence and factors associated with BC diagnosis delay. In this study, educational status, occupation, and residency also were announced as important factors regarding BC diagnosis delay.

In (56–58, 62, 63), and (60), different types of LR have been employed to assess the association between various sociodemographic and clinical factors and the risk of BC diagnosis delay.

The main strength of this paper is utilizing four different machine learning methods and comparing the outcomes, whereas in other papers, only one or two methods were used. We used a wide range of variables that might influence the rate of progression of BC. Recruiting participants who visited the biggest referral center in the southern part of Iran makes the results generalizable to the city’s population.

The generalizability of the data might be pointed out as a limitation of this study, as the data were collected from one referral center in the south of Iran (no other parts of the country); however, this center is considered the source point for diagnosis and treatment of patients; also, some factors that could have affected the outcome, such as BMI and menopause status, had to be omitted due to the missing data. Future studies can consider a larger dataset that is collected from different centers in different cities to achieve more generalized outcomes and build more reliable models.

## 5 Conclusion

Early diagnosis plays a significant role in increasing the survival rate of BC patients. The diagnosis of cancer by pathologists is costly, and the outcome might vary greatly depending on the pathological process. Also, due to the human brain’s limited ability to integrate large amounts of data, the accuracy of the diagnosis cannot be guaranteed, and it is impossible to avoid misdiagnosis. Artificial intelligence models are superb at handling large amounts of data. With the use of machine learning, which is a subset of artificial intelligence, an accurate and quick diagnosis of BC is possible. Machine learning techniques suggest that women with an urban residency who got married or had their first child at an age older than 30 and those who are nulliparous are at a higher risk of diagnosis delay, and it is necessary to be educated about BC symptoms and self-breast examination.

## Data availability statement

The original contributions presented in the study are included in the article/supplementary material. Further inquiries can be directed to the corresponding authors.

## Ethics statement

The ethics code was obtained from the ethics committee of Shiraz University of Medical Sciences.

## Author contributions

Conceptualization: SD, KS, and RM. Data: MD-N, MF, and SD. Methodology: SD, MD-N, SS, RM, and KS. Formal analysis and investigation: MD-N, SS, KS, and RM Writing—original draft preparation: SD. Writing—review and editing: MF, MD-N, RM, and MM. All authors contributed to the article and approved the submitted version.
